# Advantageous Uses of Mass Spectrometry for the Quantification of Proteins

**DOI:** 10.1155/2013/219452

**Published:** 2013-01-08

**Authors:** John E. Hale

**Affiliations:** Hale Biochemical Consulting, 6341 Wyatt Lane, Klamath Falls, OR 97601, USA

## Abstract

Quantitative protein measurements by mass spectrometry have gained wide acceptance in research settings. However, clinical uptake of mass spectrometric protein assays has not followed suit. In part, this is due to the long-standing acceptance by regulatory agencies of immunological assays such as ELISA assays. In most cases, ELISAs provide highly accurate, sensitive, relatively inexpensive, and simple assays for many analytes. The barrier to acceptance of mass spectrometry in these situations will remain high. However, mass spectrometry provides solutions to certain protein measurements that are difficult, if not impossible, to accomplish by immunological methods. Cases where mass spectrometry can provide solutions to difficult assay development include distinguishing between very closely related protein species and monitoring biological and analytical variability due to sample handling and very high multiplexing capacity. This paper will highlight several examples where mass spectrometry has made certain protein measurements possible where immunological techniques have had a great difficulty.

## 1. Introduction

Quantitative mass spectrometry of proteins has evolved dramatically over the last decade. Early methods involved labeling proteins with reagents enriched in stable isotopes in order to introduce mass tags into proteins of interest for relative quantification of proteins [1, 2]. These reagents have been refined over the years and have found widespread use in the form of the ITRAQ reagent [3]. Metabolic labeling of proteins with stable isotope-enriched amino acids has also been used as a technique for the relative quantification of proteins in cell culture systems [4]. Additionally, “label-free” methods have been developed for relative quantification of proteins in complex mixtures [5, 6]. As mentioned, these methods were developed for relative quantification of proteins, that is, comparing two or more samples and determining whether levels of proteins increased or decreased in response to some perturbation. Isotopically labeled peptides have been used as standards for the absolute quantification of proteins in complex mixtures. Variations of this approach include the AQUA and SISCAPA methods [7, 8]. Among the advantages of these techniques is that a quantitative assay may be developed for a given protein without the need for an antibody [7]. Alternatively an antibody to a synthetic peptide may be used [8]. This greatly simplifies the development of assays from immunological formats, such as ELISA, where well-characterized antibodies are needed. However, immunological assays are considered the standard type of assay when quantifying proteins in clinical settings. This is due, in part, to the sensitivity, accuracy, high through put, and relative simplicity of the technology. The implementation of mass spectrometric assays has been slow in this arena since mass spectrometric assays have not provided a clear advantage over ELISA assays. There are certain cases where mass spectrometry is able quantify proteins that are very difficult or impossible to measure by immunological methods. Some of these cases include assaying individual protein isoforms in the presence of all isoforms, measurement of specifically modified proteins, quantification of panels of proteins, and quantification of processed forms of proteins in biological samples. In addition, quantitative mass spectrometric assays may have advantageous dynamic range. While outside the scope of this review, using different combinations of protein analysis and mass spectrometers, dynamic ranges (defined as the lowest level of protein measurable relative to the most abundant protein in a sample) can vary from 1 to 2 orders of magnitude to 4-5 orders of magnitude [9]. There are many different types of mass spectrometry available and in some cases, different types of mass spectrometers may be used to develop assays for the same analyte. Selection of a mass spectrometer may depend on several factors including the necessary throughput, complexity of the sample, and method of introduction of the sample to the mass spectrometer. MALDI-Tof mass spectrometry may be adapted to very high throughput applications, as data acquisition requires only a few seconds; however, it may require more sample cleanup prior to mass analysis. Electrospray techniques may be interfaced with liquid chromatographic sample introduction to add an additional sample simplification step prior to mass analysis. Coupling to triple quadrupole or ion-trap instruments allows for the development of assays that can detect the analyte ion directly through extracted ion chromatography (XIC) or that can detect a fragment ion or ions after MS/MS analysis (known as selected reaction monitoring, SRM or multiple reaction monitoring, MRM). In cases where sample complexity is low, XIC may be sufficient for assay development. If complexity is higher, SRM or MRM techniques add an additional level of specificity for a given ion and can reduce overlap with contaminating ions. Finally, in cases of very high sample complexity very high resolution detectors such as FTICR or Orbitrap instruments may be used to isolate very narrow mass ranges for subsequent analysis, thereby reducing contaminating ion overlap. This paper will discuss specific examples of these situations and compare the efficiency and ease of use between mass spectrometry and immunological methods.

## 2. Measurement of Protein Isoforms

Quantification of protein isoforms can be a significant challenge for immunological assay development. Isoforms may result from substitution of only a few amino acids in a protein sequence. Thus, for immunological assay development, highly specific antibodies need to be developed. Mass spectrometry can detect substitutions of single amino acids in proteins and may be used to quantify individual protein isoforms in mixtures. 

Using class-specific isolation methods, quantitative assays for protein isoforms may be developed without the need for any antibody. An example of this approach is an assay for the three common apolipoprotein E isoforms, Apo E2, E3, and E4. Amino acid substitutions in positions 112 and 158 of the protein sequence define the isoforms (Table 1). Apo E4 is associated with increased risk for Alzheimer's disease [10] and Apo E polymorphism has associations with cardiovascular disease [11]. Thus, quantification of the different isoforms is important in understanding the role of Apo E in the various disease states. Immunological methods require the use of different combinations of antibodies for each amino acid substitution and also require multiple assays be performed on a single sample. For instance, an Apo E2 carrier may not be distinguished from an ApoE3/E4 carrier using antibodies recognizing the epitopes with C112 and C158 alone. Antibodies recognizing epitopes with R112 and R158 would need to be included meaning that four separate assays would need to be developed. Recently, a mass spectrometric assay was developed that can measure all three ApoE isoforms simultaneously [12]. By using a lipoprotein absorbant, no antibody was needed to isolate the proteins. Following tryptic digestion, ion-trap-based multiple reaction monitoring assays were designed for the peptides LGADMEDVC_112_GR, LGADMEDVR_112_, LAVYQAGAR, and C_158_LAVYQAGAR. By quantitatively measuring each of these peptides, the concentrations of each isoform could be calculated. This was accomplished with the inclusion of Apo E2 and E4 standards that were metabolically labeled with 13C leucine. This strategy allowed for the quantification of both total Apo E and specific isotope concentrations from a single sample measurement. 

Protein isoforms may be isolated using polyclonal or pan-protein antibodies. The protein transthyretin (TTR) provides an example of this approach. Transthyretin is a 127 amino acid protein that tetramerizes and functions as a carrier of T4 and retinol (by binding to retinol binding protein) [13]. Mutations in the protein are associated with a condition known as Familial Transthyretin Amyloidosis [14]. More than one hundred mutant forms of TTR are known and many of these are associated with pathological familial amyloid diseases [15]. At least fifteen nephropathic mutants have been discovered, with a single amino acid substitution of V to M at position 30 as the most common mutation [16]. Diagnosis consists of a combination of DNA testing and IEF analysis [17]. DNA testing can detect amino acid mutations but cannot detect posttranslational modifications (PTMs). IEF may detect some PTMs but may also miss some. Transthyretin monomer is small enough to be directly measured by MALDI-Tof mass spectrometry. Mass spectrometric methods have been developed that involve immunocapture of transthyretin from plasma with polyclonal antibodies followed by MALDI MS analysis of the isolated protein [18]. This technique simultaneously detects amino acid variants, as well as, PTMs, thus negating the need for two tests. By including internal standards (such as isotopically labeled transthyretin), this method may be made quantitative. A more recent refinement of this method incorporated a standard curve in a reference sample yielding a linear working range of thirtyfold [19]. This assay compared very well with an ELISA for TTR but had the advantage of being able to assay multiple TTR variants in a single sample in a high throughput manner which the ELISA cannot do.

Therapeutic monoclonal antibodies (mAb) provide an important challenge for quantification of protein isoforms. In order to monitor drug levels and monitor clearance rates during a therapeutic treatment, assays must measure the levels of a single antibody isoform in the background of host antibody, which consists of thousands of proteins with very high levels of sequence identity with the therapeutic mAb. Immunological methods typically utilize antigenic capture or anti-idiotypic antibodies to isolate the specific antibody of interest. Mass spectrometric methods negate the need for these isolation steps due to the presence of unique peptide sequences in the complementarity determining regions (CDRs) of the antibodies. Individual monoclonal antibodies may be quantified in complex mixtures using SRM or MRM of specific peptides from enzymatic digestions of plasma samples [20–22]. In one example of this approach, a specific, unique peptide from a human monoclonal antibody could be detected in tryptic digests of human plasma [20]. The detectability of this peptide could be increased at least threefold by a simple two-step solid phase extraction procedure. The assay had a linear range of more than three orders of magnitude and had very good accuracy and precision values in a three-day validation analysis. This assay performed as well or better than an ELISA assay for a monoclonal antibody in a rat pharmacodynamic study. Inclusion of isotopically labeled protein standards allowed for the measurement of absolute levels of mAbs in serum samples [21]. Spiking serum samples with mAb labeled with stable isotopes controls for losses incurred during sample processing and cleanup, since the labeled mAb behaves identically to the unlabeled mAb. Studies monitoring a human mAb spiked into a total IgG fraction from human serum demonstrated that peptides from an individual mAb may be detected by extracted ion chromatography at levels five orders of magnitude below the total IgG concentration [23]. Thus, by isolating the total IgG fraction using protein G, individual antibodies present at 0.01% of the total may be detected. This extends the potential of this approach to the measurement of disease-associated antibodies in biological samples and allows for these assays to be developed rapidly without the need for specific antibody reagents.

Perhaps the most extreme example of isomeric protein quantification is the measurement of epigenetic modification of histones [24–26]. Epigenetics is the study of the regulation of gene expression by cellular modification of DNA and proteins. A major component of this regulation is the various posttranslational modifications that occur on the DNA binding proteins, the histones. This “histone code” controls access to DNA thus regulating gene expression [27]. Modifications that occur include mono-, di-, and tri-methylation of lysine, acetylation of lysine, mono- and di-methylation of arginine, phosphorylation of serine, threonine, and tyrosine. There are literally millions of combinations in which these different modifications may occur, making antibody methods impossible for quantitative analysis. LC/MS/MS methods offer the only practical method of quantifying these modifications and assessing their importance in cellular differentiation and maintenance of phenotype. Studies of the N-terminal 23 amino acid tail of histone 4 using nanoflow-LC coupled to high resolution mass spectrometry indicated multiple different modified forms. Inclusion of ETD MS/MS allowed assignment of positional modifications and revealed the presence of seventy-four different forms of the N-terminal tail. A label-free quantification technique allows for the assignment of the relative abundance of these forms and makes possible the quantification of changes in the histone epigenome in response to cellular perturbations [26].

## 3. Measurement of Biologically Processed Proteins

In addition to isoforms and posttranslational modification, protein heterogeneity may arise from degradation by proteases, esterases, phosphatases, deacetylases, and many other metabolic enzymes. Degradation of proteins may occur as a part of normal metabolism but may also be artifactual, the result of enzymatic activation during sample collection or processing. Mass spectrometry provides a means to distinguish biological from artifactual processing. Labeling of proteins with stable isotopes creates a standard that can be distinguished by mass spectrometry. These protein standards are chemically and biologically indistinguishable from the unlabeled protein. Introduction of the labeled standard into a biological system prior to sample processing then allows one to monitor the effects of sample handling on the heterogeneity of the protein. An example of this is the peptide hormone, ghrelin. Ghrelin is a 28 amino acid peptide that has an octanoic acid moiety attached to a serine residue at position 3 in the peptide sequence [28]. This modification is essential for the biological activity of ghrelin, which includes increased appetite [29] and increased insulin secretion [30]. The octanoic acid group is easily removed by esterases, which may be activated during sample collection [31, 32]. This may lead to a wide variation in measured levels of octonyl and des octonyl ghrelin. Distinguishing biological from artifactual heterogeneity of ghrelin is difficult if not impossible by immunological methods. By adding 13C labeled ghrelin standards (octonylated and unoctonylated) into sample collection tubes, deacylation upon collection of serum samples may be monitored in real time [33]. Using this assay, it was found that immediate acidification of blood samples was a highly effective method for preserving the acylated version of ghrelin and allowed for much more accurate measurement of its true biological levels.

Another important example of biological heterogeneity of a protein entity is the amyloid beta peptide (A-beta). The association of A-beta with Alzheimer's disease (AD) is well known [34], and its role as a causative agent has recently been strengthened [35]. A-beta exists in multiple forms, the most prevalent of which are 40 and 42 amino acid long peptides. These peptides derive from the transmembrane domain of the Alzheimer's precursor protein (APP) through the action of different proteases. The 42 amino acid peptide (containing two additional C-terminal amino acids from APP) is hydrophobic and aggregates to form plaques in the brain that are the hallmark of AD [34]. A-beta peptides are also present in cerebrospinal fluid, with the 40 amino acid version being more prevalent [36]. Characterization of A-beta from isolated CSF and from brain extracts has demonstrated the presence of additional heterogeneity in the peptide. A-beta immunoprecipitated from CSF exists in multiple C-terminally truncated forms [36]. Conversely, A-beta peptides extracted from brain tissue exhibit heterogeneity at the N-terminus [37]. The differences in the localization of these isoforms may have important implications for the biology of AD and may also provide important biomarkers for therapeutic drug development. The major A-beta isoforms (40 and 42 amino acid versions) have been typically measured by ELISA [38]. ELISA measurement of the multiple forms of the peptides by ELISA is made difficult by the need to develop antibodies that will specifically recognize peptides that differ only by truncation of individual amino acids. Cross reactivity is likely to be high and many antibodies must be developed. Mass spectrometric assays have been developed for A-beta peptides that involve immunoprecipitation with an antibody that cross reacts with multiple A-beta isoforms. Stable isotope labeled peptides, spiked into biological samples, provide standards to control for peptide recovery and to monitor peptide stability through the assay procedure. One such assay employing LC/ESI/MS/MS was developed to simultaneously monitor A-beta 1–40 and 1–42. [39] This stable isotope dilution assay utilized 15N labeled A-beta 1–40 and 1–42 standards spiked into cerebrospinal fluid (CSF) from Alzheimer's disease patients and healthy controls followed by immunoprecipitation with an antibody to the midregion of A-beta [40]. Immunoprecipitated A-beta 1–40 and 1–42 were separated by reversed phase HPLC under basic conditions and sprayed into a linear ion trap mass spectrometer. Peptides were fragmented by MS/MS and quantified by selected reaction monitoring. The labeled internal standards allowed for absolute quantitative analysis, as well as, monitoring sample processing effects. This assay had good sensitivity and strong correlation with the ELISA for A-beta 1–42. 

Another assay employing MALDI-Tof mass spectrometry was used to quantify seven different forms of the A-beta peptide in CSF from individuals with Alzheimer's disease and healthy volunteers [41]. The performance of this assay resulted in a quantitative range of nearly two orders of magnitude. Comparison of the mass spectrometric assay to the ELISA assays for the 40 and the 42 amino acid versions of the A-beta peptide showed very good correlations (0.95 and 0.88, resp.). No N- or C-terminal processing of the isotopically labeled peptides was observed ruling out artifactual production of the various forms during sample preparation for the assay. 

Yet another multiplex quantitative assay was developed using immunoprecipitation followed by ion trap LCMS analysis [42]. By using an antibody to the mid-region of A-beta, ten different versions of the peptide could be isolated. After positive identification by a combination of accurate mass and MS/MS analyses, quantification was achieved for each of the peptides by extracting ion current for the two most abundant charge states and normalizing the peaks to an isotopically labeled A-beta internal standard. The assays were linear over at least a tenfold concentration range with very low relative standard deviation. This assay has been shown to be useful for *in vitro* drug efficacy and mechanism of action studies [42].

## 4. Conclusion

Several different examples of the advantageous use of mass spectrometry for the assay of closely related protein species have been cited in this paper. Of course there are many additional examples, such as the use of mass spectrometry to quantify protein phosphorylation. The ability of mass spectrometry to accurately distinguish different isoforms or modified forms of proteins, even in mixtures, provides added dimensions in the design of quantitative strategies and simplifies multiplex assay development. Isotopically labeled protein or peptide standards allow for absolute quantification and also provide methods for monitoring stability of analytes throughout sample processing steps involved in preparation for measurement. The sensitivity of mass spectrometers is very high and limitations on the sensitivity of quantitative assays are more often related to the dynamic range of proteins in a sample. Better sample simplification procedures are often the key to increased sensitivity of mass spectrometric protein assays. Perhaps the major limitation to mass-spectrometry-based protein quantification is throughput. Immunological assays can be performed in 96- or 384-well format. Plate readers can measure readouts of entire plates in batch mode. On the other hand, mass spectrometers must analyze samples one at a time. Even under very fast analytical conditions, such as MALDI-Tof analysis, each sample will require a few seconds to process. Future developments in multiplexed mass spectrometric detectors may help to address this limitation [43].

## Figures and Tables

**Table 1:** Amino acid substitutions in Apolipoprotein E Isoforms.

Isoform	Amino acid at position 112	Amino acid at position 158
Apo E2	Cys	Cys
Apo E3	Cys	Arg
Apo E4	Arg	Cys
